# Residual Monomer Elution from Permanent Crown Materials at Different Storage Times

**DOI:** 10.3390/polym18192355

**Published:** 2026-09-27

**Authors:** Aybüke Kara, Ayşe Rençber Kızılkaya, Güler Yıldırım, Arzu Karatepe

**Affiliations:** 1 Department of Prosthodontics, Faculty of Dentistry, Fırat University, 23119 Elazığ, Turkey; arencber@firat.edu.tr (A.R.K.); gyavcu@firat.edu.tr (G.Y.); 2 Department of Chemistry, Faculty of Science, Fırat University, 23119 Elazığ, Turkey; akaratepe23a@gmail.com

**Keywords:** 3D printing, CAD/CAM, permanent resin, residual monomer, high-performance liquid chromatography, storage time

## Abstract

Three-dimensional (3D)-printed permanent crown resins are increasingly used in restorative dentistry, but their residual monomer elution compared with computer-aided design/computer-aided manufacturing (CAD/CAM) materials remains insufficiently characterized. This in vitro study compared residual monomer elution from three permanent crown materials at different storage times. A CAD/CAM hybrid ceramic (GC Cerasmart 270) and two 3D-printed permanent crown resins (Graphy Tera Harz and Saremco CrownTec) were evaluated. Specimens (12 × 14 × 2 mm; *n* = 5 per material) were stored in 75% ethanol, and the extraction solutions were analyzed by high-performance liquid chromatography (HPLC) on days 1 and 30. The eluted amounts of Bis-GMA, TEGDMA, UDMA, and HEMA were quantified and analyzed using repeated-measures ANOVA, Kruskal–Wallis, and Wilcoxon signed-rank tests (*p* < 0.05). Material, time, and their interaction were significant for Bis-GMA (*p* < 0.001). Material differences were also significant for TEGDMA and UDMA (*p* = 0.002), whereas the time-dependent increases in TEGDMA, UDMA, and HEMA did not reach statistical significance. Graphy showed the highest Bis-GMA and UDMA elution, Saremco the highest TEGDMA and HEMA elution, and Cerasmart the lowest values for all monomers. Both material characteristics and storage time should therefore be considered when evaluating residual monomer elution.

## 1. Introduction

With advances in digital dentistry, three-dimensional (3D) printing, also known as additive manufacturing technology, has come into widespread use in the fabrication of indirect restorations, alongside computer-aided design and computer-aided manufacturing (CAD/CAM) systems [1,2]. Initially limited to the fabrication of interim restorations, the scope of 3D printing technology has expanded over time, and resin-based materials that can be used for the fabrication of various prosthetic restorations, including permanent crown materials, have now been developed [3]. The rapid and standardized production enabled by 3D printing technology, along with reduced material waste and the ability to fabricate complex restoration geometries, has increased interest in its use for the fabrication of permanent prosthetic restorations. In addition, the reproducibility of digital designs when needed and the direct integration of the manufacturing process into the digital workflow have positioned 3D printing as an alternative method for the fabrication of permanent restorations [3,4].

Resin-based permanent crown materials are indirect restorative materials composed of a methacrylate-based organic resin matrix and inorganic filler particles [5,6]. Because not all monomers are converted into the polymer network during the polymerization reaction, a certain amount of residual monomer may remain within the material structure, and these monomers can be eluted into the surrounding environment over time through diffusion [7]. Unlike provisional restorations, these materials are intended to remain in the oral environment for extended periods, raising concerns regarding the prolonged potential for residual monomers to interact with oral tissues and surrounding fluids. Therefore, with the increasing use of 3D-printed resins for permanent restorations, determining the amount of residual monomers released from these materials and changes in their elution over time has become important for evaluating their biological safety [8]. Bis-GMA, TEGDMA, UDMA, and HEMA are among the most frequently investigated residual monomers in resin-based dental materials, and the elution of these compounds is of importance for the evaluation of the biocompatibility of these materials [9,10,11].

Subtractively manufactured CAD/CAM blocks are polymerized industrially under controlled high-temperature and high-pressure conditions, which yields a high degree of conversion, whereas 3D-printed resins are photopolymerized layer by layer and depend on post-curing procedures to complete polymerization; these differences in manufacturing may therefore result in different residual monomer elution profiles [5,7]. In addition, the amount of residual monomer eluted from resin-based dental materials is influenced by numerous factors, including the chemical composition of the material, monomer structure, filler content, degree of conversion, post-polymerization procedures, and storage conditions [8,12]. In particular, storage time and the extraction medium are among the main variables determining the diffusion of residual monomers, and the amount eluted may change over time [8,13]. Therefore, the evaluation of the residual monomer elution of different resin materials under standardized conditions is important for comparing the biological performance of these materials [12,14].

Studies evaluating residual monomer elution in dentistry have mostly focused on direct resin composites, CAD/CAM materials, interim restorative materials, and 3D-printed denture base resins [15,16,17]. Recent studies on 3D-printed permanent crown resins have mainly compared them with provisional resins [8,12] or with subtractively manufactured materials [13,14]; however, comparative data on the residual monomer elution of individual monomers from commercially available permanent crown materials across different storage times, and on how this elution changes over time, remain scarce. Therefore, a comparative evaluation of the residual monomers eluted from resin-based permanent crown materials fabricated by different manufacturing methods under standardized experimental conditions is needed.

The aim of this in vitro study was to comparatively evaluate the amounts of residual monomers eluted from a subtractively manufactured CAD/CAM permanent crown material and two different additively manufactured 3D-printed permanent crown materials at different storage times. The null hypotheses of the study were that (1) there would be no significant difference in residual monomer elution among the permanent crown materials fabricated by different manufacturing methods, and (2) storage time would not significantly affect the amount of residual monomer eluted.

## 2. Materials and Methods

### 2.1. Materials and Study Design

**Figure 1:**
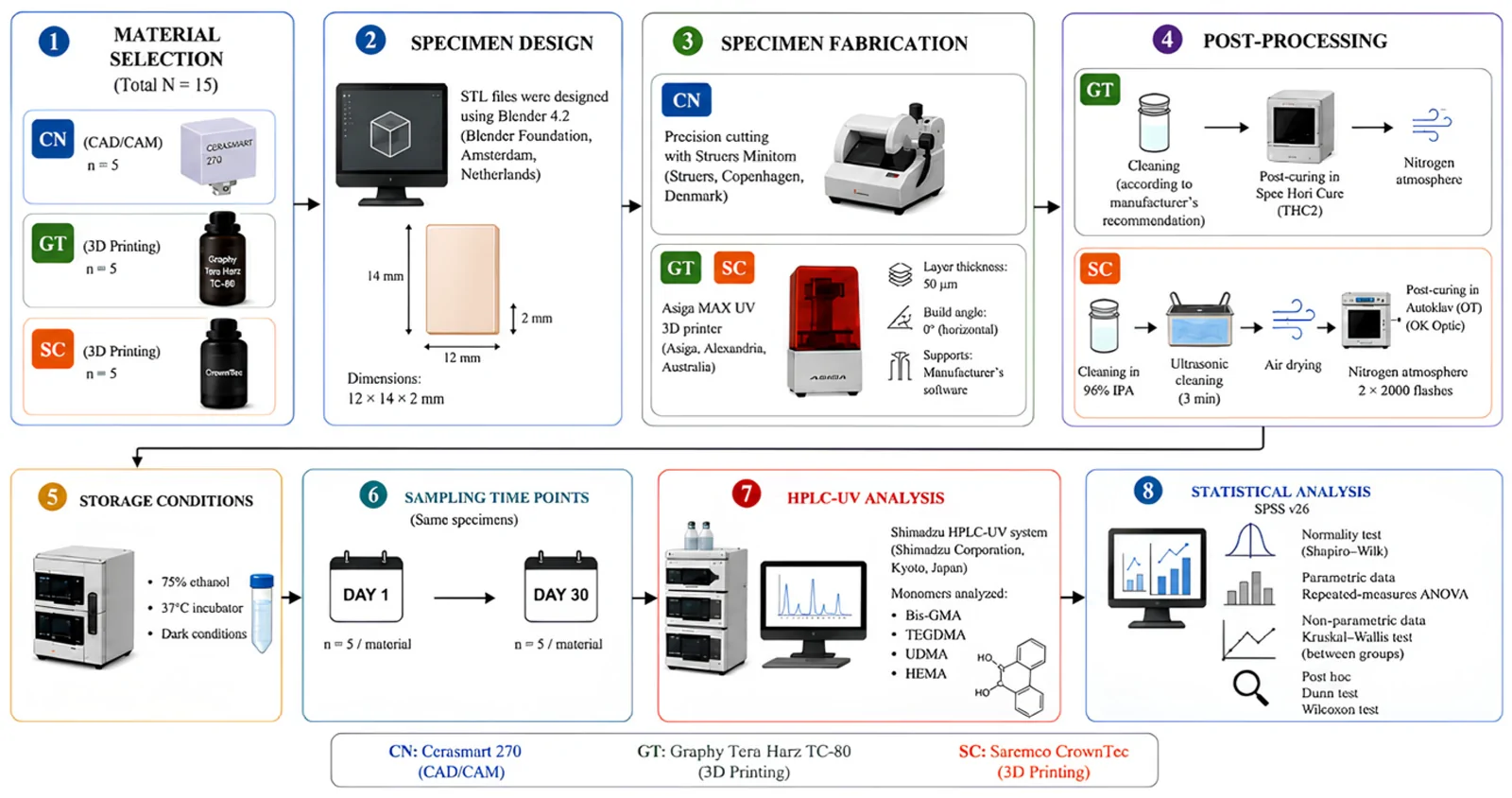
Experimental design and workflow of the study.

Three different permanent crown materials were evaluated in this in vitro study: Cerasmart 270 nanohybrid ceramic block (CN; GC Corporation, Tokyo, Japan), Graphy Tera Harz TC-80 permanent crown resin (GT; Graphy Inc., Seoul, Republic of Korea), and Saremco CrownTec permanent crown resin (SC; Saremco Dental AG, Rebstein, Switzerland). CN was included as the reference CAD/CAM material fabricated by subtractive manufacturing, whereas GT and SC were included as permanent crown resins produced by additive manufacturing. The manufacturer-reported technical characteristics of the materials used in this study are presented in Table 1. The experimental design and workflow of the study are summarized in Figure 1.

### 2.2. Specimen Fabrication

The STL (Standard Tessellation Language) files of the 12 × 14 × 2 mm specimens were designed using Blender 4.2 software (Blender Foundation, Amsterdam, The Netherlands). The GT and SC specimens were fabricated with an Asiga MAX UV 3D printer (Asiga, Alexandria, Australia) at a layer thickness of 50 µm. During printing, the specimens were positioned horizontally (0°) on the build platform, and the support structures generated by the manufacturer’s software were used. After printing, the GT specimens were cleaned in accordance with the manufacturer’s recommendations and subjected to post-polymerization under a nitrogen atmosphere in a Tera Harz Cure unit (THC2; Graphy Inc., Seoul, Republic of Korea). The SC specimens were cleaned with 96% isopropyl alcohol, ultrasonically cleaned for 3 min, air-dried, and post-polymerized in an NK Optik Otoflash G171 unit (NK Optik, Baierbrunn, Germany) under a nitrogen atmosphere with 2 × 2000 flashes. The GC Cerasmart 270 blocks (12 × 14 × 18 mm) were sectioned into 2-mm-thick specimens using a precision cutting machine (Struers Minitom; Struers, Copenhagen, Denmark).

### 2.3. Extraction Protocol

A power analysis based on a large expected effect size (f = 1.0, α = 0.05, power = 0.80, three groups) determined that a minimum of five specimens per material (total *n* = 15) was required to detect between-material differences in residual monomer elution. The power analysis was designed for the primary between-material comparison and was not specifically powered to detect within-material changes between the two extraction time points. Five specimens were prepared for each material (*n* = 5), yielding a total of 15 specimens. Each specimen was placed individually in a 20-mL glass vial containing 5 mL of extraction solution. The extraction solution consisted of a 75% ethanol/25% water mixture prepared with 99.9% ethanol (Isolab, Eschau, Germany) and distilled water. The extraction procedure was conducted with reference to ISO 10993-12:2021 [18]. The total exposed surface area of each specimen was approximately 4.4 cm^2^, corresponding to an initial surface area-to-extraction volume ratio of 0.88 cm^2^/mL. Although this ratio was lower than that recommended by ISO 10993-12 [18], the extraction volume of 5 mL was selected to ensure complete immersion of each specimen and to provide sufficient solution for sampling at both time points. The vials were incubated at 37 °C in the dark. At Day 1, a 1 mL aliquot was withdrawn from each vial, transferred into a labeled glass microvial, and stored at 4 °C until HPLC analysis. The withdrawn volume was not replaced; therefore, each specimen remained in the remaining 4 mL of extraction solution until Day 30, corresponding to a surface area-to-volume ratio of 1.10 cm^2^/mL. At Day 30, a further 1 mL aliquot was collected and stored under the same conditions. To account for the reduction in extraction volume and the amount of monomer removed during the first sampling, the cumulative amount released at Day 30 was calculated as M_30_ = (C_1_ × 1 mL) + (C_30_ × 4 mL), where C_1_ represents the concentration measured in the Day 1 aliquot and C_30_ represents the concentration measured in the remaining extraction solution at Day 30. The cumulative amount was subsequently normalized to the initial extraction volume of 5 mL and reported in ppm. These cumulative values were used in the statistical analyses.

Following completion of the 30-day extraction period, the specimens were subjected to a qualitative macroscopic visual assessment. Representative photographs of the exposed specimens and unexposed specimens of the same materials were obtained under comparable background and lighting conditions. The assessment focused on visible cracking, fracture, gross deformation, and obvious surface alterations. No quantitative measurements of mass, dimensions, color, or surface roughness were performed.

### 2.4. HPLC-UV Analysis

HPLC-UV analyses were performed using a Shimadzu high-performance liquid chromatography system (Shimadzu Corporation, Kyoto, Japan) equipped with an SPD-M20A photodiode-array detector (PDA; Shimadzu Corporation, Kyoto, Japan). Chromatographic separation was achieved using a reversed-phase C18 analytical column (250 × 4.6 mm, 5 µm particle size; GL Sciences Inc., Torrance, CA, USA). Prior to the analyses, standard solutions of Bis-GMA (Sigma-Aldrich, St. Louis, MO, USA), TEGDMA (BLD Pharmatech Ltd., Shanghai, China), UDMA (Sigma-Aldrich, St. Louis, MO, USA), and HEMA (BLD Pharmatech Ltd., Shanghai, China) were prepared in the range of 0.63–50 ppm for calibration of the instrument (R^2^ > 0.99). Following calibration, each sample result was individually reviewed. Samples producing detector responses below the calibration range at the standard injection volume were reanalyzed using an increased injection volume, whereas samples producing responses above the calibration range were appropriately diluted and reanalyzed. The final concentrations were back-calculated by accounting for the modified injection volume or dilution factor. Therefore, the reported concentrations below 0.63 ppm and above 50 ppm represent back-calculated concentrations in the original samples and were not determined by direct extrapolation beyond the calibration curve. The approximate retention times were 5.4 min for Bis-GMA, 3.8 min for TEGDMA, 4.4 min for UDMA, and 2.9 min for HEMA. The analyses were performed under isocratic conditions with a column temperature of 40 °C and a detection wavelength of 210 nm. Different wavelengths were evaluated using a PDA detector, and 210 nm was selected as the analytical wavelength because it provided the most suitable absorbance for all of the monomers investigated. Chromatographic suitability was monitored throughout the analyses on the basis of retention times and peak separation. The mobile phase consisted of a 3:2:2:3 (*v*/*v*) mixture of ethanol, acetonitrile, methanol, and distilled water. The flow rate was set at 1 mL/min, and the standard injection volume was 20 µL. The total analysis time was 10 min. Residual monomer concentrations (ppm) were calculated by applying the obtained peak areas to the corresponding calibration curves, with correction for any modification in injection volume or sample dilution, and were used in the statistical analyses.

### 2.5. Statistical Analysis

Statistical analyses were performed using IBM SPSS Statistics for Windows, Version 26.0 (IBM Corp., Armonk, NY, USA). Distributional assumptions were not uniformly satisfied across the Material × Time cells. Bis-GMA was normally distributed in all six cells (Shapiro–Wilk *p* ≥ 0.144) and was therefore analyzed with a mixed two-way repeated-measures ANOVA. TEGDMA, UDMA and HEMA each contained at least one non-normal cell (minimum Shapiro–Wilk *p* = 0.004, <0.001 and 0.002, respectively) and were consequently analyzed with nonparametric procedures. Homogeneity of variance was satisfied for all monomers except Bis-GMA at 1 day (Levene W = 4.773, *p* = 0.030); because variances were homogeneous both at 30 days (*p* = 0.122) and for the difference scores (*p* = 0.076), and because the design was balanced (*n* = 5 per cell), the ANOVA was retained.

Between-material comparisons at each time point were performed with one-way analysis for Bis-GMA within the mixed-model framework and with Kruskal–Wallis tests for TEGDMA, UDMA, and HEMA, followed by Dunn’s post-hoc test with Bonferroni adjustment. Within-material comparisons between 1 day and 30 days were performed with paired *t*-tests for Bis-GMA and with Wilcoxon signed-rank tests for the nonparametric monomers, with *p* values adjusted using the Holm procedure. Effect sizes were reported as partial eta squared (ηp^2^), epsilon squared (ε^2^), Cohen’s d, and the matched-pairs rank–biserial correlation (r), as appropriate. The level of statistical significance was set at *p* < 0.05.

## 3. Results

Descriptive statistics for all Material × Time combinations are presented in Table 2.

### 3.1. Bis-GMA

Mixed repeated-measures ANOVA revealed significant main effects of material (F(2, 12) = 365.663, *p* < 0.001, ηp^2^ = 0.984) and time (F(1, 12) = 565.317, *p* < 0.001, ηp^2^ = 0.979), together with a significant material × time interaction (F(2, 12) = 245.532, *p* < 0.001, ηp^2^ = 0.976), indicating that the magnitude of the increase in Bis-GMA release between 1 day and 30 days was material-dependent (Table 3). Graphy showed the highest Bis-GMA release at both time points, rising from 15.129 ± 2.637 at 1 day to 87.147 ± 9.002 at 30 days, followed by Saremco (7.614 ± 0.361 to 30.404 ± 0.919). Cerasmart showed the lowest values throughout (1.750 ± 0.436 to 2.660 ± 1.439). Paired comparisons confirmed significant time-related increases for Graphy (*p* < 0.001, d = 8.20) and Saremco (*p* < 0.001, d = 27.81), whereas the increase observed for Cerasmart did not reach significance (*p* = 0.315, d = 0.51) (Table 4).

### 3.2. TEGDMA

Kruskal–Wallis tests indicated significant differences among materials at both 1 day (H(2) = 12.590, *p* = 0.002, ε^2^ = 0.882) and 30 days (H(2) = 12.590, *p* = 0.002, ε^2^ = 0.882). Dunn–Bonferroni post-hoc comparisons showed that Saremco released significantly more TEGDMA than Cerasmart at both time points (adjusted *p* = 0.001), whereas the Cerasmart–Graphy and Graphy–Saremco contrasts did not remain significant after correction (adjusted *p* = 0.228 for all). Median TEGDMA release increased from 0.193 to 0.841 for Cerasmart, from 2.609 to 9.798 for Graphy, and from 11.586 to 106.934 for Saremco. Despite these consistent descriptive increases, none of the within-material Wilcoxon signed-rank comparisons reached significance after Holm correction (adjusted *p* = 0.188 for all three materials).

### 3.3. UDMA

Material differences were significant at both time points (1 day: H(2) = 12.613, *p* = 0.002, ε^2^ = 0.884; 30 days: H(2) = 12.106, *p* = 0.002, ε^2^ = 0.842). Post-hoc analysis identified Graphy as releasing significantly more UDMA than Cerasmart at 1 day (adjusted *p* = 0.001) and at 30 days (adjusted *p* = 0.002); no other pairwise contrast survived correction. Median UDMA release rose from 1.647 to 2.131 for Cerasmart, from 5.319 to 10.667 for Graphy, and from 3.684 to 4.041 for Saremco, although, as for TEGDMA, the within-material time contrasts were not significant after Holm correction (adjusted *p* = 0.188 for all materials).

### 3.4. HEMA

Material differences were not significant at 1 day (H(2) = 3.807, *p* = 0.149, ε^2^ = 0.151) but became significant at 30 days (H(2) = 6.909, *p* = 0.032, ε^2^ = 0.409). Post-hoc comparisons showed that Saremco released significantly more HEMA than Cerasmart at 30 days (adjusted *p* = 0.026), while the remaining contrasts were non-significant. Median HEMA release increased from 1.214 to 2.680 for Cerasmart, from 1.417 to 3.009 for Graphy, and from 1.657 to 4.645 for Saremco; none of these within-material increases reached significance after Holm correction (adjusted *p* = 0.188 for all materials).

**Figure 2:**
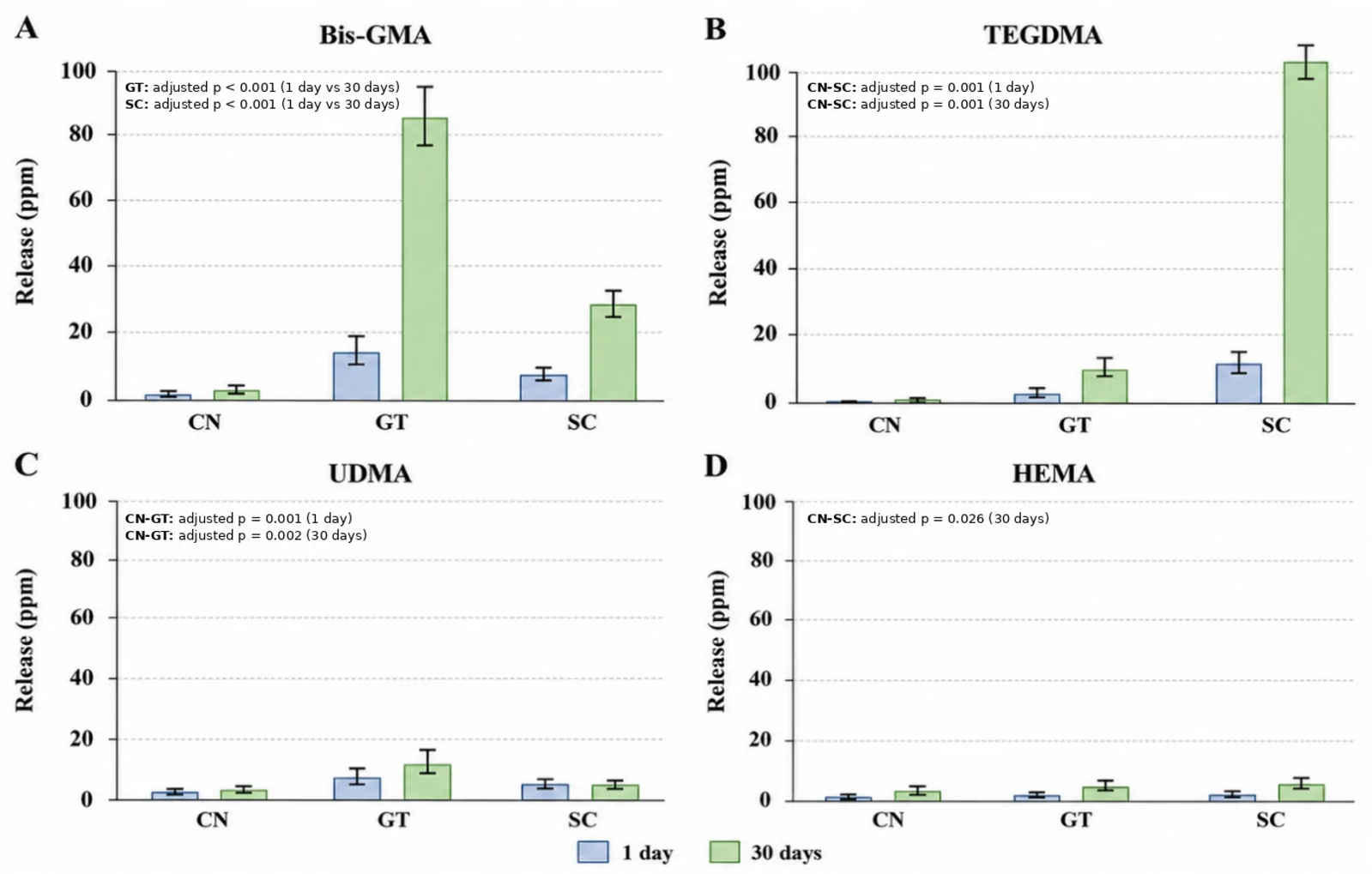
Residual monomer release (ppm) from the permanent crown materials after 1 day and 30 days of storage. (**A**) Bis-GMA, presented as mean ± standard deviation (SD), (**B**) TEGDMA, (**C**) UDMA, and (**D**) HEMA, presented as median with interquartile range (IQR). Blue bars represent the 1-day measurements, whereas green bars represent the 30-day measurements. Error bars represent SD in panel (**A**) and IQR in panels (**B**–**D**). CN, Cerasmart 270; GT, Graphy Tera Harz TC-80; SC, Saremco CrownTec. Only statistically significant comparisons are displayed. For Bis-GMA, only within-material time comparisons are displayed. Within-material time comparisons for Bis-GMA were performed using paired *t*-tests with Holm adjustment, whereas between-material comparisons for TEGDMA, UDMA, and HEMA were performed using Dunn’s post-hoc test with Bonferroni adjustment.

The amounts of residual monomers eluted from the different permanent crown materials after 1 day and 30 days of storage are shown in Figure 2.

**Figure 3:**
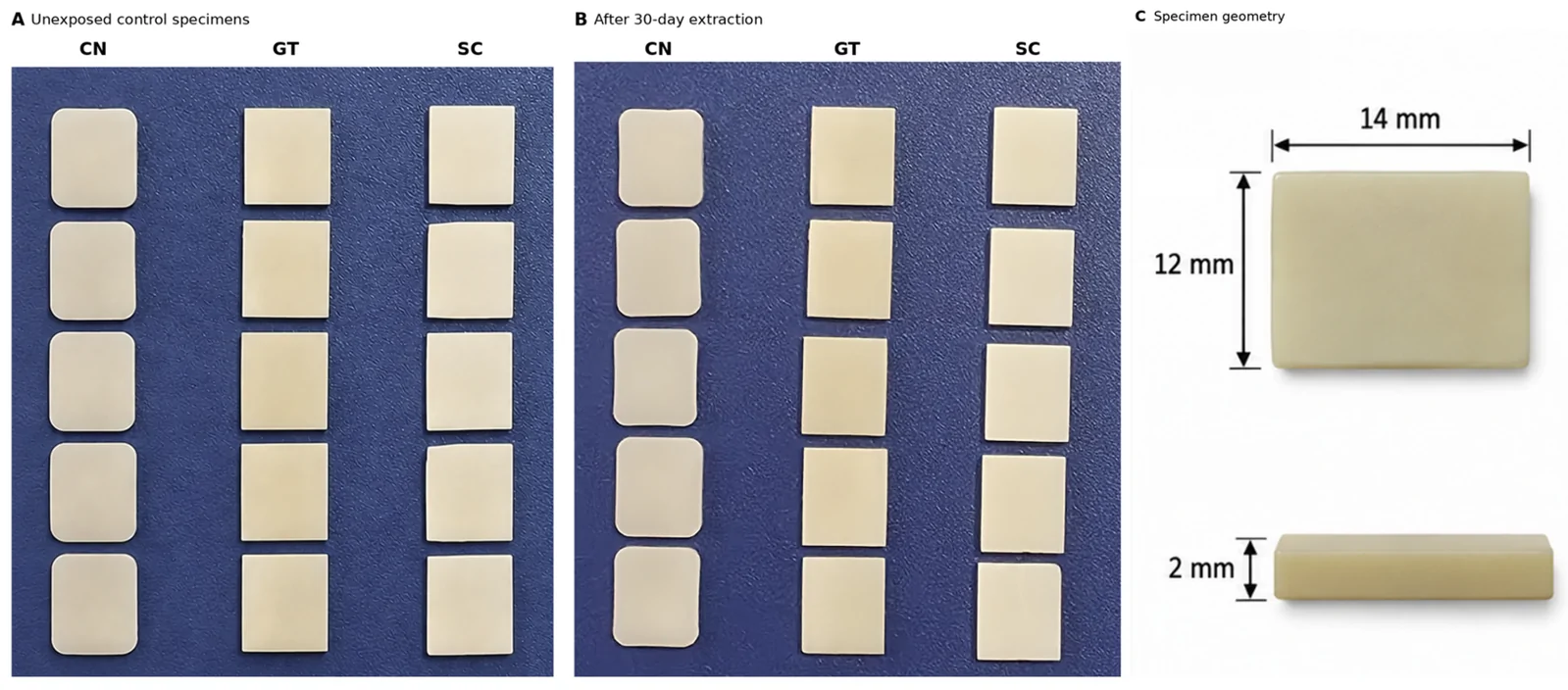
Representative macroscopic photographs of (**A**) unexposed control specimens and (**B**) specimens after 30 days of extraction in 75% ethanol/25% water. The columns show CN (Cerasmart 270), GT (Graphy Tera Harz TC-80), and SC (Saremco CrownTec). Control and exposed specimens are separate specimens, not before-and-after images of the same sample. (**C**) Schematic of the specimen geometry (12 × 14 × 2 mm; not to scale).

Representative macroscopic photographs of the unexposed and 30-day-exposed specimens are presented in Figure 3. Qualitative visual examination revealed no obvious macroscopic cracking, fracture, or gross deformation in any of the materials after the extraction period. Subtle changes in color, dimensions, mass, or surface characteristics could not be determined by visual assessment alone.

## 4. Discussion

The principal finding of this in vitro study was that the subtractively manufactured CAD/CAM hybrid ceramic material exhibited the lowest elution values for all monomers investigated, whereas markedly higher residual monomer elution was observed for the two additively manufactured 3D-printed permanent crown materials. This finding suggests that residual monomer elution may be associated with both the manufacturing approach and the material-specific chemical composition. However, because only one CAD/CAM material and two 3D-printed materials with different formulations were evaluated, the independent effects of manufacturing method and material composition cannot be distinguished. Moreover, residual monomer elution varied not only according to the material type but also according to the monomer type and storage time. Although storage time tended to increase monomer elution, this effect was not of the same magnitude for all monomers and materials. These findings indicate that residual monomer elution cannot be explained by a single factor and that multiple variables, including the chemical structure of the material, the manufacturing method, and the storage time, should be evaluated together. Accordingly, the first null hypothesis was rejected, as significant differences in residual monomer elution were detected among the materials. The second null hypothesis was partially rejected, because a statistically significant time-dependent increase was observed only for Bis-GMA in the Graphy and Saremco groups, whereas the changes in the other monomers over the storage period did not reach statistical significance.

The lower residual monomer elution observed for the CAD/CAM hybrid ceramic material, GC Cerasmart 270, may be related to the combined effects of its industrial polymerization conditions and material-specific composition. However, this finding cannot be attributed solely to the manufacturing method. CAD/CAM blocks are polymerized under industrial conditions at high temperature and pressure, resulting in a higher degree of conversion, a more homogeneous polymer network, and lower porosity, which has been reported to reduce the amount of unreacted residual monomer [14,19,20]. In contrast, the final properties of 3D-printed resins are affected by numerous process-specific variables, such as the printing procedure, material formulation, washing, and post-polymerization protocol [21]. These process- and formulation-related factors may have contributed to the higher residual monomer elution observed for the two 3D-printed materials evaluated in this study. Regarding the individual monomers, the most pronounced difference among the materials was observed in Bis-GMA elution, and Bis-GMA elution increased significantly with storage time, particularly in the 3D-printed materials; this may be explained by the diffusion of unreacted Bis-GMA monomers remaining within the polymer network into the extraction medium over time. The increasing trends observed for TEGDMA, UDMA, and HEMA in all materials during the storage period can likewise be regarded as expected findings, although these changes did not reach statistical significance; this may be attributable to the limited sample size, the differences in chemical composition among the materials, and the reduction in statistical power resulting from the Holm correction applied for multiple comparisons. Similarly, the significant difference among the materials for HEMA only at 30 days suggests that the diffusion of this monomer into the extraction medium continues over time. Taken together, although a trend toward higher elution at 30 days compared with 1 day was observed for all monomers, this increase was statistically significant only for Bis-GMA in the Graphy and Saremco groups, and the absence of a significant change in the Cerasmart group over the storage period is compatible with a relatively stable polymer network. Nevertheless, because the evaluated materials differed in both manufacturing approach and chemical composition, this finding cannot be attributed specifically to the industrial polymerization process.

The findings of our study are consistent with previous reports in the literature. Atasoy et al. [13] reported that additively manufactured permanent restorative materials showed higher monomer elution than CAD/CAM materials and, in line with our TEGDMA findings, also observed a storage time-dependent increase in TEGDMA elution from additively manufactured permanent crown resins [13]. Similarly, Prawatvatchara et al. [8] reported that different 3D-printed permanent resins exhibited markedly different monomer profiles despite the use of similar manufacturing technology, which is in agreement with the differences observed between the Graphy and Saremco materials in our study. Intarak et al. [12] also reported that monomer elution from different 3D-printed dental resins was substantially affected by the polymerization efficiency and resin formulation of the material. Furthermore, the low monomer elution observed in the Cerasmart group is consistent with the findings of Yıldırım et al. [14], who reported lower monomer elution from subtractively manufactured indirect restorative materials; the same authors also evaluated monomer elution together with cell adhesion and cell viability in indirect restorative materials fabricated by additive, subtractive, and conventional methods, highlighting the importance of considering residual monomer elution together with biological performance [14].

When the structure–property relationships are considered, residual monomer elution from resin-based materials depends not only on the initial degree of conversion but also on the density of the polymer network, the cross-linking structure, and the monomer composition [7,12]. In materials with lower polymerization efficiency, more free monomers may remain within the polymer network, and these monomers can be gradually eluted into the medium during storage as the solvent penetrates the polymer network [7]. The intrinsic molecular characteristics of Bis-GMA may also have contributed to the observed behavior; its bulky aromatic backbone, high molecular weight, and high viscosity restrict molecular mobility during polymerization, which can limit the degree of conversion and leave a larger fraction of unreacted Bis-GMA entrapped within the polymer network, while solvent-induced swelling of the resin matrix facilitates the subsequent diffusion of these molecules during storage. In contrast, TEGDMA is known to pass more easily from the polymer network into the surrounding environment owing to its low molecular weight and high diffusion capacity, whereas UDMA and HEMA elution are similarly affected by factors such as the resin composition and polymerization efficiency of the material. The differences observed among the materials may therefore have originated not only from the resin composition but also from differences in the resin matrix, filler content, and photoinitiator systems; the significant time-dependent increase in Bis-GMA only in the Graphy and Saremco groups suggests that the polymer network structure and resin composition of these materials may have characteristics that facilitate long-term monomer diffusion. However, because detailed quantitative compositional information is not disclosed by the manufacturers, this relationship cannot be directly established and should be interpreted with caution. Moreover, these explanations represent plausible, literature-supported mechanisms rather than mechanisms directly demonstrated in the present study. Because only two extraction time points were evaluated and the degree of conversion, solvent sorption, polymer-network structure, and diffusion coefficients were not measured, the exact release kinetics and the relative contribution of each mechanism could not be determined.

One of the strengths of this study is that the residual monomer elution of permanent crown materials fabricated with CAD/CAM and 3D printing technologies was comparatively evaluated under the same standardized experimental conditions, using an extraction protocol conducted with reference to ISO 10993-12, quantitative HPLC-UV analyses, and the evaluation of Bis-GMA, TEGDMA, UDMA, and HEMA in the same specimens at different storage times. Nevertheless, this study has some limitations. First, the study was conducted under in vitro conditions, and the 75% ethanol extraction medium was selected for accelerated aging with reference to ISO 10993-12; therefore, the monomer elution values obtained may not exactly reflect the actual amounts eluted in the oral environment under clinical conditions. Moreover, 75% ethanol represents an aggressive extraction medium that can promote resin-matrix swelling and monomer release. Therefore, the concentrations measured in the present study should be interpreted as representing a worst-case extraction scenario rather than actual intraoral exposure, where monomer concentrations would be expected to be substantially lower. Although the cumulative calculation accounted for the amount of monomer removed during Day 1 sampling, the extraction volume decreased from 5 to 4 mL after sampling, increasing the surface area-to-volume ratio from 0.88 to 1.10 cm^2^/mL. This change may have influenced the subsequent release equilibrium; therefore, comparisons between the two extraction time points should be interpreted with caution. Although the macroscopic visual assessment did not reveal obvious cracking, fracture, or gross deformation after extraction, this qualitative observation cannot exclude subtle changes in mass, dimensions, color, surface roughness, or microstructure. Quantitative physical measurements and microscopic surface characterization, including SEM analysis, were not performed and should be included in future studies. In addition, only three different permanent crown materials and four common residual monomers were evaluated, and the investigation of different materials or other monomers may yield different results. Moreover, because only one CAD/CAM material and two 3D-printed materials were included and these materials differed in chemical composition, the effects of manufacturing method and material formulation on monomer elution could not be evaluated independently. Therefore, the findings should be interpreted as material-specific comparisons rather than as general differences between subtractive and additive manufacturing methods. Furthermore, the sample size was relatively small (*n* = 5 per material), which, together with the statistical corrections applied for multiple comparisons, may have limited the statistical power to detect within-material differences over the storage period. Accordingly, the non-significant time effects should not be interpreted as evidence of an absence of temporal change but rather as inconclusive findings that require confirmation in studies with larger sample sizes. Monomer elution was evaluated for up to 30 days, and longer-term elution behavior was not investigated. Moreover, because parameters related to monomer elution, such as the degree of conversion, cytotoxicity, and mechanical properties, were not evaluated, the relationship between the observed residual monomer elution and these parameters could not be directly established. Accordingly, the measured monomer concentrations should be interpreted as chemical elution data and not as direct evidence of cytotoxicity, biological harm, or clinical risk. The results are valid only for the commercial materials evaluated in this study and should not be generalized to all restorative materials. Furthermore, each 3D-printed material was processed using a single manufacturer-recommended washing and post-curing protocol. Because these parameters were not varied independently, their specific effects on residual monomer elution could not be distinguished from the effects of material composition. Future studies should systematically vary washing duration, washing medium, post-curing time, and post-curing atmosphere within the same material to determine their individual effects on monomer release.

It should also be emphasized that the demonstration of residual monomer elution alone does not imply the presence of a biological or clinical risk. Future studies should therefore investigate residual monomer elution together with biological parameters, such as cytotoxicity and cell viability, as well as the degree of conversion and mechanical properties, in order to clarify the relationships among these parameters. Such an integrated approach would help determine whether the material- and monomer-specific elution profiles observed in the present study are associated with measurable biological effects. In addition, the use of clinically more relevant extraction media such as artificial saliva, the evaluation of longer aging periods, and the inclusion of a wider range of commercial materials and monomers would contribute to a more comprehensive assessment of the clinical potential of 3D-printed permanent crown materials.

## 5. Conclusions

1. Residual monomer elution from permanent crown materials varied depending on the material type, monomer type, and storage time.

2. Among the materials evaluated, Cerasmart 270 exhibited the lowest residual monomer elution, whereas higher elution values were observed for Graphy Tera Harz TC-80 and Saremco CrownTec.

3. Both material characteristics and storage time should be considered together when evaluating residual monomer elution.

4. These results are limited to in vitro study conditions, and further studies reflecting clinical conditions are needed.

## Figures and Tables

**Table 1:** Manufacturer-reported characteristics of the permanent crown materials used in this study.

Permanent Crown Materials	Manufacturer	Composition	Printing System Compatibility	Recommended Post-Curing
Cerasmart 270 (CN)	GC Corporation, Tokyo, Japan	Bis-MEPP, UDMA, dimethacrylate, silica (20 nm), barium glass (300 nm), 71 wt% inorganic fillers	CAD/CAM milling system	Not applicable
Saremco CrownTec (SC)	Saremco Dental AG, Rebstein, Switzerland	Esterification products of 4,4′-isopropylidiphenol; ethoxylated and 2-methylprop-2enoic acid; silanized dental glass; pyrogenic silica; initiators	Asiga MAX UV	Otoflash G171 (NK Optik)
Graphy Tera Harz (GT)	Graphy Inc., Seoul, Republic of Korea	Urethane acrylate oligomer, bisphenol A ethoxylate dimethacrylate, 2-HEMA, diphenyl (2,4,6-trimethylbenzoyl) phosphine oxide, and additives	Asiga MAX UV	Tera Harz Cure (THC2)

**Table 2:** Descriptive statistics for monomer release by material and storage time.

Monomer	Material	Time	n	Mean ± SD	Median (IQR)
Bis-GMA	Cerasmart	1 day	5	1.750 ± 0.436	1.872 (1.780–1.931)
		30 days	5	2.660 ± 1.439	2.982 (1.256–3.473)
	Graphy	1 day	5	15.129 ± 2.637	15.183 (14.123–16.348)
		30 days	5	87.147 ± 9.002	90.987 (84.835–93.512)
	Saremco	1 day	5	7.614 ± 0.361	7.641 (7.353–7.861)
		30 days	5	30.404 ± 0.919	30.301 (29.887–31.072)
TEGDMA	Cerasmart	1 day	5	0.364 ± 0.257	0.193 (0.181–0.633)
		30 days	5	0.814 ± 0.108	0.841 (0.738–0.888)
	Graphy	1 day	5	2.369 ± 1.458	2.609 (0.946–3.116)
		30 days	5	10.150 ± 2.904	9.798 (8.167–9.931)
	Saremco	1 day	5	12.742 ± 3.197	11.586 (11.066–14.995)
		30 days	5	107.640 ± 5.016	106.934 (104.336–111.297)
UDMA	Cerasmart	1 day	5	1.641 ± 0.141	1.647 (1.631–1.647)
		30 days	5	2.488 ± 0.816	2.131 (2.116–2.138)
	Graphy	1 day	5	5.927 ± 1.784	5.319 (5.319–5.995)
		30 days	5	11.474 ± 1.690	10.667 (10.662–12.588)
	Saremco	1 day	5	3.511 ± 0.483	3.684 (3.170–3.766)
		30 days	5	4.300 ± 0.818	4.041 (3.843–4.886)
HEMA	Cerasmart	1 day	5	1.205 ± 0.524	1.214 (1.119–1.290)
		30 days	5	2.570 ± 0.395	2.680 (2.459–2.875)
	Graphy	1 day	5	1.405 ± 0.150	1.417 (1.393–1.476)
		30 days	5	3.842 ± 2.327	3.009 (2.788–3.997)
	Saremco	1 day	5	1.766 ± 0.529	1.657 (1.434–2.152)
		30 days	5	4.525 ± 0.605	4.645 (4.050–4.744)

Values are expressed in ppm. IQR, interquartile range (Q1–Q3); SD, standard deviation. *n* = 5 specimens per cell.

**Table 3:** Between-material comparisons of monomer release at each time point.

Monomer	Time	Test	Test Statistic	*p*	Effect Size	Significant Pairwise Comparisons (Adjusted *p*)
Bis-GMA	1 day/30 days	RM-ANOVA (material)	F(2,12) = 365.663	<0.001	ηp^2^ = 0.984	See Table 4 (interaction)
TEGDMA	1 day	Kruskal–Wallis	H(2) = 12.590	0.002	ε^2^ = 0.882	Saremco > Cerasmart (0.001)
	30 days	Kruskal–Wallis	H(2) = 12.590	0.002	ε^2^ = 0.882	Saremco > Cerasmart (0.001)
UDMA	1 day	Kruskal–Wallis	H(2) = 12.613	0.002	ε^2^ = 0.884	Graphy > Cerasmart (0.001)
	30 days	Kruskal–Wallis	H(2) = 12.106	0.002	ε^2^ = 0.842	Graphy > Cerasmart (0.002)
HEMA	1 day	Kruskal–Wallis	H(2) = 3.807	0.149	ε^2^ = 0.151	None
	30 days	Kruskal–Wallis	H(2) = 6.909	0.032	ε^2^ = 0.409	Saremco > Cerasmart (0.026)

RM-ANOVA, mixed two-way repeated-measures analysis of variance; ηp^2^, partial eta squared; ε^2^, epsilon squared. Pairwise contrasts for nonparametric analyses were performed with Dunn’s test and Bonferroni adjustment. For Bis-GMA, the time main effect (F(1, 12) = 565.317, *p* < 0.001, ηp^2^ = 0.979) and the material × time interaction (F(2, 12) = 245.532, *p* < 0.001, ηp^2^ = 0.976) were also significant.

**Table 4:** Within-material comparison of monomer release between 1 day and 30 days.

Monomer	Material	Test	1 Day	30 Days	Statistic	Adjusted *p*	Effect Size
Bis-GMA	Cerasmart	Paired *t*-test	1.750 ± 0.436	2.660 ± 1.439	t(4) = −1.148	0.315	d = 0.51
	Graphy	Paired *t*-test	15.129 ± 2.637	87.147 ± 9.002	t(4) = −18.325	<0.001	d = 8.20
	Saremco	Paired *t*-test	7.614 ± 0.361	30.404 ± 0.919	t(4) = −62.173	<0.001	d = 27.81
TEGDMA	Cerasmart	Wilcoxon	0.193	0.841	W = 0.000	0.188	r = 0.83
	Graphy	Wilcoxon	2.609	9.798	W = 0.000	0.188	r = 0.83
	Saremco	Wilcoxon	11.586	106.934	W = 0.000	0.188	r = 0.83
UDMA	Cerasmart	Wilcoxon	1.647	2.131	W = 0.000	0.188	r = 0.83
	Graphy	Wilcoxon	5.319	10.667	W = 0.000	0.188	r = 0.83
	Saremco	Wilcoxon	3.684	4.041	W = 1.000	0.188	r = 0.69
HEMA	Cerasmart	Wilcoxon	1.214	2.680	W = 0.000	0.188	r = 0.83
	Graphy	Wilcoxon	1.417	3.009	W = 0.000	0.188	r = 0.83
	Saremco	Wilcoxon	1.657	4.645	W = 0.000	0.188	r = 0.83

Bis-GMA values are mean ± SD; values for the nonparametric monomers are medians. *p* values were adjusted with the Holm procedure. d, Cohen’s d; r, matched-pairs rank–biserial correlation. For Wilcoxon signed-rank tests with *n* = 5, the smallest attainable two-sided exact *p* value is 0.0625, so no within-material contrast can reach significance after correction for multiple comparisons.

## Data Availability

The raw data supporting the conclusions of this article are contained within the article. Further inquiries can be made available by the authors on request.

## Abbreviations

The following abbreviations are used in this manuscript:

3D	three-dimensional
ANOVA	analysis of variance
Bis-GMA	bisphenol A-glycidyl methacrylate
CAD/CAM	computer-aided design/computer-aided manufacturing
HEMA	2-hydroxyethyl methacrylate
HPLC	high-performance liquid chromatography
IQR	interquartile range
SD	standard deviation
STL	Standard Tessellation Language
TEGDMA	triethylene glycol dimethacrylate
UDMA	urethane dimethacrylate
